# Treatment with the NK1 Antagonist Emend Reduces Blood Brain Barrier Dysfunction and Edema Formation in an Experimental Model of Brain Tumors

**DOI:** 10.1371/journal.pone.0097002

**Published:** 2014-05-12

**Authors:** Elizabeth Harford-Wright, Kate M. Lewis, Mounir N. Ghabriel, Robert Vink

**Affiliations:** Adelaide Centre for Neuroscience Research, School of Medical Sciences, University of Adelaide, Adelaide, South Australia, Australia; University Hospital of Navarra, Spain

## Abstract

The neuropeptide substance P (SP) has been implicated in the disruption of the blood-brain barrier (BBB) and development of cerebral edema in acute brain injury. Cerebral edema accumulates rapidly around brain tumors and has been linked to several tumor-associated deficits. Currently, the standard treatment for peritumoral edema is the corticosteroid dexamethasone, prolonged use of which is associated with a number of deleterious side effects. As SP is reported to increase in many cancer types, this study examined whether SP plays a role in the genesis of brain peritumoral edema. A-375 human melanoma cells were injected into the right striatum of male Balb/c nude mice to induce brain tumor growth, with culture medium injected in animals serving as controls. At 2, 3 or 4 weeks following tumor cell inoculation, non-treated animals were perfusion fixed for immunohistochemical detection of Albumin, SP and NK1 receptor. A further subgroup of animals was treated with a daily injection of the NK1 antagonist Emend (3 mg/kg), dexamethasone (8 mg/kg) or saline vehicle at 3 weeks post-inoculation. Animals were sacrificed a week later to determine BBB permeability using Evan's Blue and brain water content. Non-treated animals demonstrated a significant increase in albumin, SP and NK1 receptor immunoreactivity in the peritumoral area as well as increased perivascular staining in the surrounding brain tissue. Brain water content and BBB permeability was significantly increased in tumor-inoculated animals when compared to controls (p<0.05). Treatment with Emend and dexamethasone reduced BBB permeability and brain water content when compared to vehicle-treated tumor-inoculated mice. The increase in peritumoral staining for both SP and the NK1 receptor, coupled with the reduction in brain water content and BBB permeability seen following treatment with the NK1 antagonist Emend, suggests that SP plays a role in the genesis of peritumoral edema, and thus warrants further investigation as a potential anti-edematous treatment.

## Introduction

Brain tumors are one of the most devastating forms of cancer, and unlike many other cancer types, their incidence is increasing. Currently within Australia, 1400 new cases of malignant brain tumors are diagnosed and approximately 1100 people die from the disease each year [1]. Astrocytomas and metastatic carcinomas are the most common types of brain tumors in adults. Metastatic brain tumors are up to 10 times more common than primary tumors of the brain [2] and frequently originate from primary carcinomas of breast, lung and colon, as well as melanomas [3]. Despite recent advances in cancer treatment, brain tumors remain inherently difficult to treat and prognosis for these patients remains extremely poor, with most individuals succumbing to the disease within months of diagnosis.

Both aggressive astrocytomas and secondary brain tumors are associated with significant edema formation, which results in substantial morbidity and mortality amongst patients. Indeed, edematous fluid can accumulate rapidly around the tumors at volumes of up to 90 ml per day [4], [5]. Due to the rigid nature of the skull, accumulation of this fluid is associated with a number of deleterious consequences and if left untreated can result in ischemia, herniation and death [6]. Brain tumor-associated edema is typically vasogenic in nature, arising from the flux of fluids across a compromised blood-brain barrier (BBB). Under normal physiological circumstances, the BBB is made up of endothelial cells joined by tight junctions. Tight junctions consist of molecules such as claudin-3, claudin-5 and claudin-12, as well as other transmembrane proteins such as occludin [7]. The disruption to the BBB surrounding tumors is thought to result from defects in these tight junctions, with abnormal expression of tight junction molecules reported to correlate with increasing malignancy [8]. Despite extensive knowledge of the structure of the BBB, molecular mechanisms for the disruption associated with brain tumor growth remains poorly understood.

Recent research has implicated substance P (SP) in the genesis of edema formation following other types of injury to the CNS [9]-[12]. SP is a neuropeptide that is released from perivascular sensory nerve fibres and preferentially binds to the NK1 receptor. Mechanical stimulation of these fibres following injury causes SP release and the subsequent development of neurogenic inflammation. Neurogenic inflammation is characterised by increased vasodilation, plasma extravasation and thus, edema formation [11]. Research has shown that there is a significant increase in SP immunoreactivity in perivascular tissue and within brain neuropil 5 hours post trauma, which corresponded with significant increases in cerebral edema [10]. Subsequent administration of an NK1 antagonist was found to attenuate this edema formation and thereby improved neurological outcome [10]. Similarly Turner *et al* (2006) showed that SP immunoreactivity was increased in the infarcted hemisphere post stroke and was associated with profound edema formation. As was observed in TBI, administration of a SP antagonist resulted in marked improvement in functional outcome following stroke [12]. Given its role in the development of edema following these insults to the CNS, and that SP expression is increased in a variety of human cancer cell lines including brain tumors [13]–[16], SP may also be a potential target for the treatment of metastatic brain tumor-associated edema [9]–[12].

However, it is not yet known whether SP levels are increased within the peri-tumoral region and if it plays a role in the genesis of tumor-associated edema. Thus, the aims of the current study were to examine the role of SP in tumor-associated edema in a model of brain tumors secondary to melanoma, and to establish whether NK1 antagonists may provide a novel alternative treatment to brain tumor edema.

## Methods

### Cell Culture

A-375 human melanoma cells were obtained from American Type Culture Collection (ATCC, CRL-1619). For all experiments, A-375 cells were placed in 150 cm^2^ flasks with 20 mL of complete culture medium (CCM) consisting of Dulbecco's modified Eagle's medium (DMEM), 10% fetal bovine serum (FBS), 5% L-glutamine and 1% penicillin and streptomycin. For tumor cell inoculation, A-375 cells were passaged once to >90% confluence before the CCM was removed from the flask and 3.5 mL of trypsin added to detach cells. Subsequently, 7.5 mL of CCM was added and the cells centrifuged for 5 minutes at 1500 RPM. Cells were re-suspended in serum free media and counted using a haemocytometer and then diluted, so that ∼10^5^ cells/5 µL were available for tumor cell inoculation.

### Animals

Animal procedures were performed in accordance with the National Health and Medical Research Council guidelines and were approved by the University of Adelaide and Institute of Medical and Veterinary Science animal ethics committees. All experimental work was performed using male Balb/C nude mice (n = 70) aged 8–10 weeks, weighing between 17 and 22 g. Animals were randomly assigned to each experimental group and were housed in specific pathogen free conditions at 24°C on a 12-hour day-night cycle. Animals were allowed access to standard rodent pellets and water *ad libitum*.

### Implantation of Tumor Cells

Tumor cell inoculation was performed using stereotaxic implantation of ∼10^5^/5 µL A-375 human melanoma cells. Briefly, mice were anaesthetised with 3% Isoflurane and placed in a stereotaxic frame, with anaesthesia maintained at 1.5% Isoflurane using a nose cone. Tumour cells were injected into the right striatum, 0-mm anterior and 2.5-mm lateral to the bregma and 3-mm deep over 10 minutes. Following injection, the needle remained in the brain for an additional 5 minutes to allow tumor cells to disperse.

### Treatment

At 3 weeks following tumor cell inoculation, animals were randomly assigned to receive treatment of saline vehicle control, the NK1 antagonist fosaprepitant diglutemide commonly known as Emend (Merck & Co) (3 mg/kg/day) or the positive control, dexamethasone (DBL) (8 mg/kg), which is the current clinical treatment for brain tumor-associated edema. Animals received a daily intraperitoneal injection of their assigned treatment for 7 days prior to euthanasia (n = 6 per Evan's Blue group, n = 5 per brain water content group). Emend was prepared by dissolving the drug in saline, with the vehicle control animals administered saline only. The dose of Emend was determined from previous studies, which have demonstrated this administration exerts central effects [17]. The dexamethasone dosage has been successfully used previously to treat experimental brain tumor associated edema [18].

### Tissue Processing

For histological analysis animals were transcardially perfused at 2, 3 or 4 weeks following tumor cell inoculation (n = 6 per group). Mice were terminally anaesthetised with an intraperitoneal injection of pentobarbital (60 mg/kg) and perfused with 10% formalin. Brains were removed, processed and embedded in paraffin wax.

### Immunohistochemistry

Assessment of immunohistochemistry was performed on six sections containing tumor or corresponding level of control per animal at 2, 3 and 4 weeks following tumor inoculation, and representative images taken. Briefly, 5 µm coronal sections were cut and stained with primary antibodies for SP (Santa Cruz; 1∶2000), NK1 receptor (ATS; 1∶8000) and Albumin (ICN Pharmaceuticals, 1∶20000). Following overnight incubation with the primary antibodies, the appropriate secondary antibody was applied (1∶250), followed by streptavidin-peroxidase complex (SPC, 1∶1000) with bound antibody then detected with 3,3′ –diaminobenzidine (DAB) and sections counterstained with haematoxylin.

### Immunohistochemical Analysis

In combination with qualitative analysis, non-subjective estimation of the level of albumin, SP and the NK1 receptor following tumour cell inoculation was performed at the 3-week time point using the colour deconvolution method [19]. Colour deconvolution determines the amount of DAB, and hence antigen within each image to allow comparisons of positive staining between and within animals. It is based on the mathematical method described by Ruifrok and Johnston [20] for separation and quantification of immunohistochemical staining by deconvolution of the colour information in RGB images. This method reliably separates the blue of haematoxylin from the brown of DAB within a section. Previous experiments have demonstrated the reliability of this method with a linear correlation demonstrated (extinction coefficient 0.96) between increasing antibody concentration and DAB weight [21].

Slides were stained using the DAB method and scanned at high resolution using the Nanozoomer (Hamamatzu, Japan). Using the associated software, images were taken of either the whole brain (albumin, NK1 receptor) or the vessels within the peritumoural region (SP). Jpeg images were then exported into ImageJ (v1.40 g NIH, Bethesda, USA) and haematoxylin and DAB stains separated using an NIH Image J macro, with background subtraction and colour correction applied to the images [19].

### Evans Blue

Permeability of the BBB was assessed using Evans blue (EB) (Sigma, E-2129), a dye that tightly binds to albumin. Under normal physiological circumstances, albumin is prevented from entering the brain neuropil by the BBB. Thus, the amount of EB dye within the brain tissue can be used to quantify BBB disruption.

At 4 weeks following tumor cell inoculation, animals were placed under isoflurane anaesthesia and injected intravenously with EB (2% EB, 2 mL/kg) 30 minutes prior to perfusion. Animals were transcardially perfused with 0.9% saline until the perfusate from the right atrium ran clear and subsequently decapitated and brains rapidly removed. The olfactory bulbs and cerebellum were discarded; the brain separated into left and right hemispheres and placed in two Eppendorf containers.

Each sample was then weighed and homogenized in 3.75 mL of PBS and 1.25 mL of 100% trichloroacetic acid solution (TCA) (Sigma, T0699) and vortexed for 2 minutes. Samples were cooled overnight at 4°C, and then centrifuged the following day for 30 minutes at 1000 g. The supernatants of each sample were subsequently measured at 620 nm using a Synergy Mx plate reader, with the amount of EB extravasation expressed as µg/g of brain tissue using a previously obtained standard curve of EB absorbance.

### Edema Measurement

In order to assess brain water content, the wet weight dry weight method was employed [11]. At 4 weeks following tumor cell inoculation, animals were re-anaesthetised with pentobarbitone and decapitated. Brains were rapidly removed from the skull, the olfactory bulbs and cerebellum discarded and the remaining brain separated into left and right hemispheres. Each hemisphere was immediately weighed for wet brain weight. Following weighing, tissue was immediately wrapped in pre-weighed, labelled aluminium foil and placed in an oven for 72 hours at 100°C. Specimens were then removed from the oven and re-weighed to obtain dry brain water weight. Brain water content in each sample was then calculated using the wet-dry method formula:
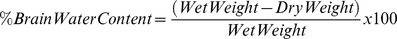



### Statistical Analysis

All data are expressed as mean±SEM and were assessed using a t-test or one or two-way ANOVA followed by Bonferonni post-tests, as appropriate. A p value of less than 0.05 was deemed significant in all experiments.

## Results

**Figure 1:**
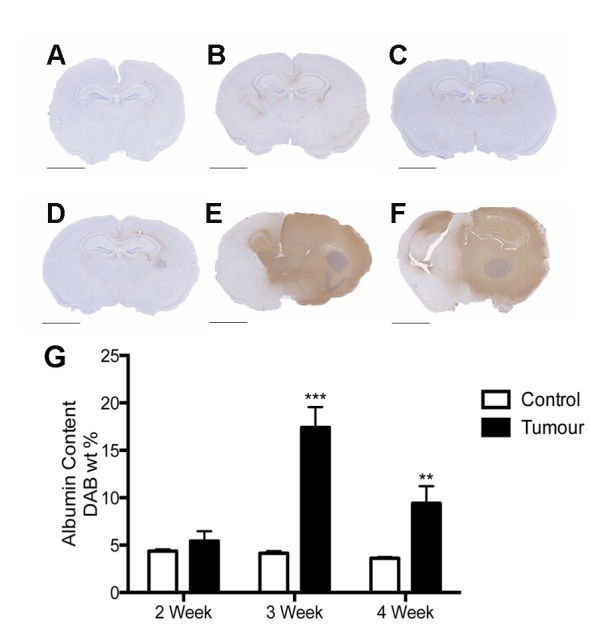
Albumin as a marker of BBB dysfunction. In control medium-injected animals, very little BBB disruption was evident at 2 weeks (A), 3 weeks (B) or 4 weeks (C) following injection. Conversely, in tumor-inoculated animals an increase in albumin staining was evident at 2 weeks (D) and extremely pronounced at 3 weeks (E) and 4 weeks (F). This was confirmed using color deconvolution, which revealed a highly significant increase in BBB disruption, as assessed by albumin staining at both 3 and 4 weeks. Scale bars = 2 mm. Image are representative of n = 6 per group. Color deconvolution analysis n = 6 per group/time point (** p<0.01, ***p<0.001 compared to controls).

Albumin is a large plasma protein that is normally prevented from entering the brain tissue by the BBB. Thus, albumin immunoreactivity was employed in this study to assess the integrity of the BBB over the 4 weeks following tumor cell inoculation (Figure 1). At 2 weeks following tumor inoculation a slight increase in albumin staining was noted in the peritumoral area. With continued tumor growth at 3 and 4 weeks post tumor inoculation, a significant increase in albumin staining occurred such that the disruption was no longer just in the immediate tumor vicinity but had spread to the contralateral side.

**Figure 2:**
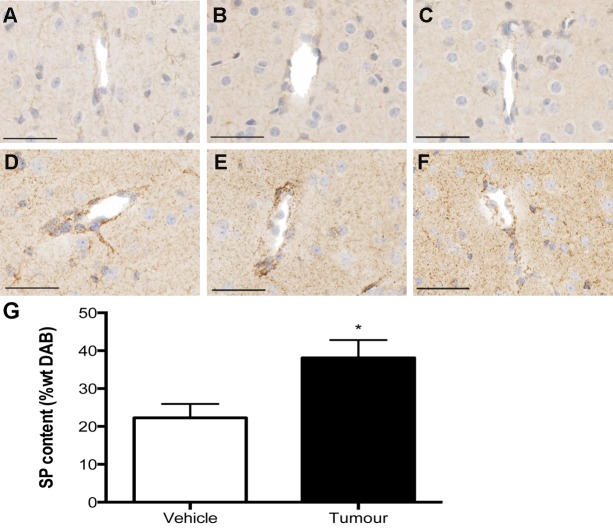
SP immunolabelled sections of peritumoral vessels. At 2 weeks (A), 3 weeks (B) and 4 weeks (C) following in culture medium injected animals, minimal SP immunoreactivity was observed in the vasculature. Following tumor cell inoculation at 2 weeks (D) a marked increase in perivascular SP staining was evident, this intensity of staining was sustained at both the 3 weeks (E) and 4 weeks (F) time points. This was confirmed by color deconvolution analysis of perivascular SP staining, which revealed a significant increase in SP immunoreactivity in tumor inoculated animals when compared to controls at 3 weeks (p = 0.039) (G). Images are representative of n = 6 per group. Scale bar = 50 µm. Color deconvolution analysis n = 6 per group (*p<0.05 compared to controls).

**Figure 3:**
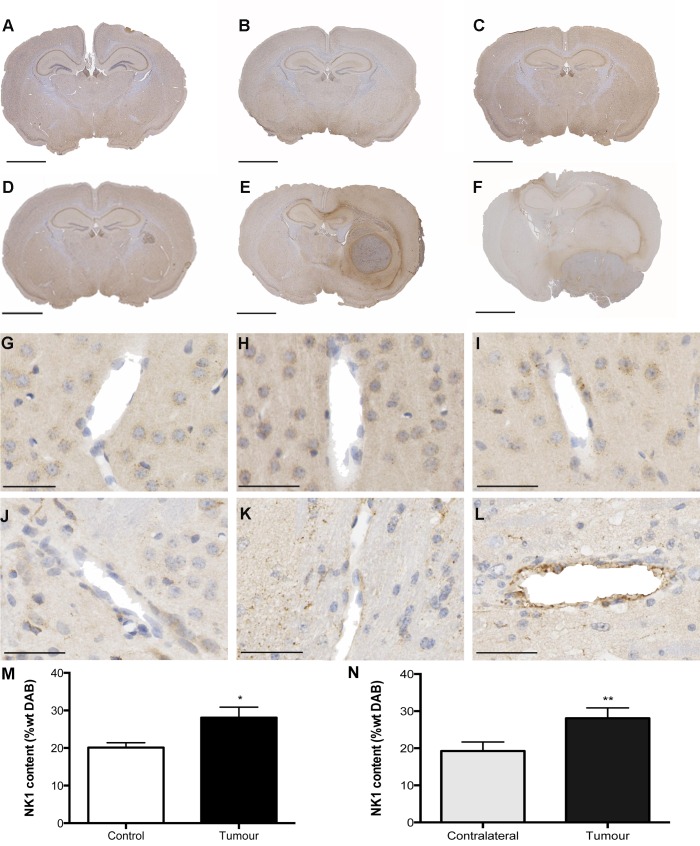
NK1 receptor immunoreactivity. A marked increase in NK1 receptor staining was observed in the area surrounding the tumor at 3 weeks (E) and 4 weeks (F) following tumor inoculation at low power when compared to the controls of the same time points (A–C). At 2 (G), 3 (H) and 4 (I) weeks following culture medium injection, there was minimal NK1 staining around the vasculature, especially when compared to the tumor inoculated animals where a moderate increase was evident at 2 (J) and 3 (K) weeks, but clearly increased at 4 weeks (L). The qualitative assessment was confirmed by color deconvolution, revealing a significant increase in overall NK1 receptor immunoreactivity when compared to controls (p = 0.0327) (M) as well as a significant increase in NK1 receptor staining in the tumor inoculated hemisphere when compared to the contralateral hemisphere of the same animals (p = 0.0086) (N). A–F Scale bars = 2 mm, G–L Scale bars = 50 µm. Images are representative of n = 6 per group. Color deconvolution analysis n = 6 per group (*p<0.05 compared to controls).

A marked increase of perivascular SP staining compared to controls was observed from 2 weeks following tumor cell inoculation and persisted until the final time point of 4 weeks (Figure 2). Due to the significant increase in BBB disruption as observed with albumin quantification, the 3 week time point was chosen for the quantification of SP and the NK1 receptor using the color deconvolution method. This confirmed the qualitative assessment, revealing a significant increase in perivascular SP staining in tumor inoculated animals compared to controls at 3 weeks. Similarly, an obvious increase in NK1 receptor staining was observed in the area surrounding the tumor such that it was apparent using low power magnification (Figure 3). This was particularly evident at the 3 weeks time point, and quantified using color deconvolution. Indeed, a significant increase in overall NK1 receptor staining was evident at 3 weeks, as well as a significant increase in NK1 receptor content in the tumor-inoculated hemisphere when compared to the contralateral hemisphere of the same animal. Only a moderate increase in perivascular NK1 receptor staining was evident at both 2 and 3 weeks following tumor cell injection, however by 4 weeks a marked increase in perivascular NK1 receptor immunoreactivity was observed (Figure 3).

**Figure 4:**
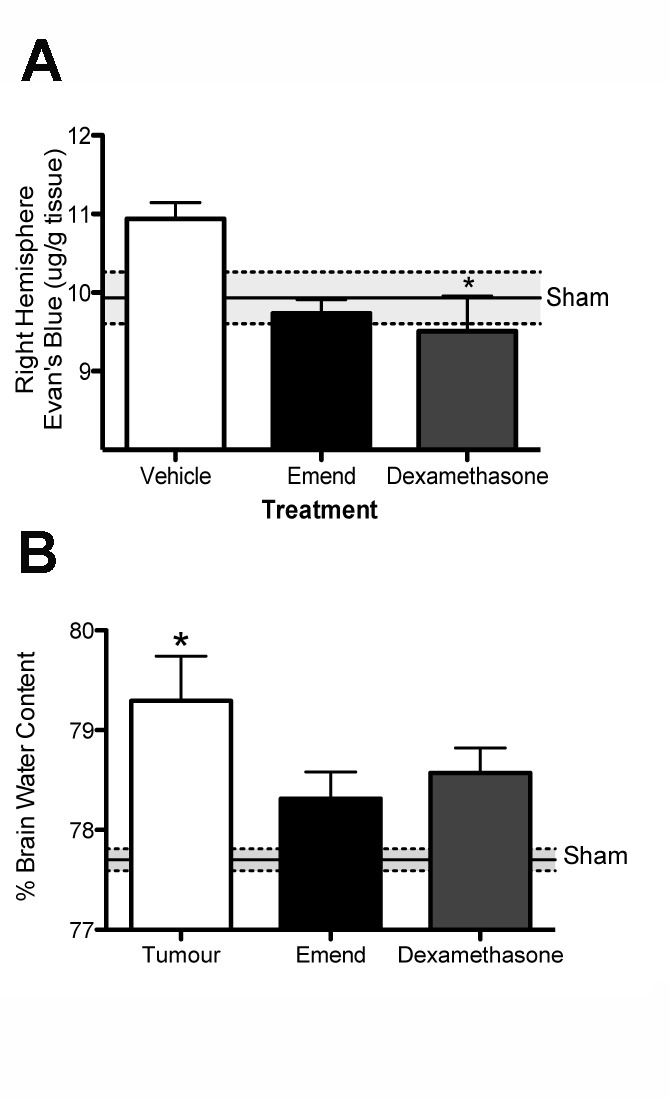
Effects of NK1 antagonist treatment on BBB permeability to Evans blue and edema formation. Using Evan's blue (EB) (A) as a marker of BBB disruption treatment with both the NK1 antagonist Emend and dexamethasone resulted in a reduction of BBB permeability back to control levels (p = 0.0237). Similarly, NK1 antagonist and dexamethasone treatment resulted in a significant reduction of brain water content (B) compared to tumor inoculated vehicle treated mice (p = 0.0188). Brain water content n = 5 per group, EB n = 6 per group (*p<0.05 compared to shams for EB, *p<0.05 compared to vehicle for WWDW).

Given that marked BBB disruption evident at 3 weeks as assessed by albumin staining, as well as corresponding significant increases in SP and NK1 receptor immunoreactivity, this time point was accordingly selected for intervention with the NK1 antagonist Emend. Following tumor cell inoculation, a significant increase in both BBB permeability and brain water content occurred compared to shams (Figure 4). Treatment with both the NK1 antagonist Emend and dexamethasone resulted in a marked reduction (p<0.05) in BBB permeability compared to vehicle-treated animals such that both these treatment groups returned to control levels by 1 week after treatment (Figure 4A). Evaluation of brain water content showed a reduction with Emend treatment, particularly within the right hemisphere. Indeed, NK1 antagonist treatment reduced brain water content to a level that was no longer significantly different compared to non-treated controls. In contrast, brain water content within the right hemisphere in vehicle (p<0.05) treated groups remained significantly increased compared to control animals.

## Discussion

Tumor-associated edema is one of the primary causes of CNS symptoms in brain tumor patients, with clinical deficits more often caused by edema than by the mass effect of the tumor itself. The potential ability of NK1 antagonists to treat edema formation associated with brain tumors is therefore of tremendous importance, especially given that the current standard treatment, dexamethasone, is associated with a number of deleterious side effects. The current study demonstrates that perivascular SP is significantly increased in the peritumoral region, which is consistent with previous studies demonstrating a role for SP in the genesis of cerebral edema in models of acute brain injury [10], [11], [22]. Administration of the NK1 antagonist Emend at 3 weeks post-inoculation resulted in a decrease in brain water content and BBB permeability such that they were no longer significantly different to sham animals within 7 days of treatment. Additionally, the NK1 antagonist was effective as the current standard treatment dexamethasone, at reducing BBB permeability and edema formation.

Stereotaxic implantation of tumor cells into the brain is the most commonly employed model of both primary and secondary brain tumors. This model reliably produces tumors of similar size and location, which allows for examination of many variables associated with brain tumors. Furthermore, it has been commonly used to detect tumor-induced changes in brain water content [23], [24]. In this study, a primary brain tumor cell line was not employed as metastatic brain tumors are more common and are associated with higher levels of edema formation [3], [25]–[28]. Immunocompromised mice are routinely used in cancer research, as the immune deficient nature of this strain allows for examination of human cancer cells in an animal model [29]. However, one limitation of the use of these animals is the translational potential of research carried out in immune deficient species. Nonetheless, the results of the present study using immunocompromised mice are consistent with previous studies performed in immune competent animals, suggesting a dominant role for SP and the NK1 receptor in neurogenic inflammation in a variety of models of CNS disease [30]–[32]. Furthermore, the pattern of SP immunoreactivity, as well as the observed decreases in BBB permeability and brain water content are consistent among these studies [10], [12], highlighting the reproducibility of these results in both immune-competent and deficient species, and thus the translational potential of SP antagonists as a therapy for cerebral edema.

In the present study, both SP and the NK1 receptor were increased in the entire tumor-inoculated hemisphere when compared to the contralateral side, indicating a possible role for SP in mediating the process of edema formation within the peritumoral region. Indeed, NK1 receptor staining was so significantly increased in the peritumoral region such that it was visible at low power. This correlated with the observed area of albumin staining, implicating SP and in the genesis of BBB breakdown and the development of cerebral edema. It was noted that the increase in both albumin and NK1 receptor staining was most pronounced at 3 weeks, and thus was chosen for quantitation. However, this peak may be more a reflection of model mortality given that a number of animals had to be euthanased due to tumor burden before the 4-week time point. Additionally, a marked increase in SP staining was observed in the vasculature surrounding the tumor at all time points, further supporting this proposed role.

The precise molecular mechanisms by which the NK1 receptor and SP are upregulated in the setting of brain tumor edema have yet to be fully elucidated. The link between inflammation and cancer has been well established, with many chronic inflammatory states associated with cancer development, as inflammation increases both mitogenesis and mutagenesis [33] [15]. Similarly, it is well known that both SP and the NK1 receptor are increased in inflammatory conditions [34], with increased expression of SP and NK1 receptor mRNA reported in response to inflammation [35]. SP induces and augments many aspects of the inflammatory response, including leukocyte activation, endothelial cell adhesion molecule expression, cytokine production and mast cell activation [36] [37]. SP may also prime polymorphonuclear cells for oxidative metabolism, increasing the production of free radicals [38]. NK1 receptors are expressed on astrocytes and are implicated in the transformation to reactive astrocytes and resultant production of inflammatory mediators [39]. Furthermore, it has been well documented that SP and the NK1 receptor are upregulated in numerous cancer types, including melanoma [14], [15], [40]–[43].

Administration of the NK1 antagonist Emend was found to profoundly reduce BBB disruption and brain water content, such that these groups were no longer significantly different to sham animals. This is consistent with previous reports implicating SP in the genesis of BBB disruption and edema formation following a number of CNS pathologies. This edematous process is thought to occur because SP induced NK1 receptor activation facilitates flux of plasma proteins from the vascular lumen, either through gaps opened between endothelial cells [44], [45] or by increasing transmembrane transport. Indeed, a recent study by Rodriguez *et al* demonstrated that SP secreted by breast cancer cells mediates their migration across the BBB. SP initiated changes in the localisation and distribution of tight junction proteins ZO-1 and Claudin-5, leading to increased permeability of the BBB, with SP antagonism reducing tumor cell migration via modulation of the BBB [46].

Additionally, the present study also found that treatment with the NK1 antagonist was as effective at reducing levels of brain edema as the current standard treatment, dexamethasone. Interestingly, Glucocorticoids have previously been shown to increase the expression of NEP and ACE in cultured cells [47], [48]. Both ACE and NEP catalyse the hydrolytic bonds of SP, cleaving the peptide at its carboxyl terminal region required for SP to bind to NK1 receptors and exert its effects [49]. Thus an increase in NEP and ACE, may lead to a decrease in SP. However, no significant changes in SP levels were observed following dexamethasone treatment in the present study, *in vitro* or *in vivo* (data not shown). Further research is required to determine the effect of dexamethasone on SP expression. Nonetheless, corticosteroids have remained the treatment of choice for tumor-associated edema for the past 40 years [50]. Stable patients with brain tumors are usually started on corticosteroids at the time of diagnosis. They are able to reduce cerebral edema in most patients by up to 70–80% and facilitate symptomatic improvement [28]. Numerous mechanisms of action have been described for corticosteroids, however, in a broad sense, corticosteroids work by reducing the permeability of a compromised BBB, thus effectively reducing edema. However, dexamethasone is associated with a large number of potentially serious side effects, the severity of which depends on the dose and duration of steroid treatment [50]. Adverse side effects include immunosuppression, hypertension, fluid retention and mood disturbances [51]. Emend is currently clinically available as an anti-emetic for chemotherapy-induced nausea, and is associated with minimal side effects.

We have previously demonstrated that Emend treatment may reduce tumor growth by prohibiting further cellular proliferation in this model of melanoma brain tumors [54]. Consequently, it is plausible that the observed reduction in BBB disruption and edema formation may be a reflection in the reduction in tumor size. It has been previously reported that the BBB is intact in small brain metastases, with angiogenesis and increased barrier permeability only associated with tumors greater than 1 mm in size [52], [53]. Thus, smaller tumors are associated with less BBB disruption and consequently less edema. Nonetheless, the results observed in this study are consistent with the role of SP in the genesis of edema in other neurological conditions, as well the SP and NK1 receptor staining correlating with the areas of increased BBB permeability. Thus, SP may potentially play multiple roles in brain tumors, and given the side effects associated with dexamethasone, warrants further investigation as a treatment for tumor associated edema.

In conclusion, the present study has demonstrated that treatment with the NK1 antagonist Emend significantly reduced tumor-associated edema and BBB disruption in an *in vivo* model of melanoma brain tumors. Furthermore, this treatment was as effective as the treatment in current clinical use, dexamethasone, thus supporting the development of therapeutics aimed at limiting the effects of SP.
